# (2,3-Di-2-pyridyl­pyrazine-κ^2^ *N* ^2^,*N* ^3^)bis­(thio­cyanato-κ*S*)palladium(II)

**DOI:** 10.1107/S1600536812000980

**Published:** 2012-01-14

**Authors:** Kwang Ha

**Affiliations:** a School of Applied Chemical Engineering, The Research Institute of Catalysis, Chonnam National University, Gwangju 500-757, Republic of Korea

## Abstract

The Pd^II^ ion in the title complex, [Pd(NCS)_2_(C_14_H_10_N_4_)], is four-coordinated in a distorted square-planar environment by the two pyridine N atoms of the chelating 2,3-di-2-pyridyl­pyrazine (dpp) ligand and two S atoms from two thio­cyanate anions. The pyridine rings are considerably inclined to the least-squares plane of the PdS_2_N_2_ unit [maximum deviation = 0.027 (1) Å], making dihedral angles of 70.3 (2) and 69.2 (1)°. The pyrazine ring is almost perpendicular to the PdS_2_N_2_ plane, with a dihedral angle of 86.3 (1)°. The thio­cyanate ligands are located on opposite sides of the PdS_2_N_2_ unit plane and are almost linear [N—C—S angles = 177.8 (6) and 178.9 (6)°]. The complex mol­ecules are stacked in columns along the *b* axis and are connected by inter­molecular C—H⋯N hydrogen bonds, forming chains along the *a* axis.

## Related literature

For related crystal structures of [Pd*X*_2_(dpp)] (*X* = Cl, Br or I), see: Ha (2011*a* ▶,*b* ▶,*c* ▶). For related Pt and Pd complexes, see: Granifo *et al.* (2000 ▶); Cai *et al.* (2009 ▶).
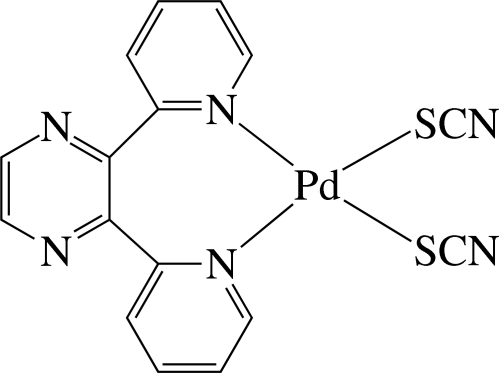


## Experimental

### 

#### Crystal data

[Pd(NCS)_2_(C_14_H_10_N_4_)]
*M* *_r_* = 456.82
Monoclinic, 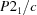

*a* = 15.8236 (11) Å
*b* = 13.5901 (9) Å
*c* = 7.9189 (6) Å
β = 102.960 (1)°
*V* = 1659.5 (2) Å^3^
*Z* = 4
Mo *K*α radiation
μ = 1.38 mm^−1^
*T* = 200 K
0.27 × 0.25 × 0.13 mm

#### Data collection

Bruker SMART 1000 CCD diffractometer
Absorption correction: multi-scan (*SADABS*; Bruker, 2000 ▶) *T*_min_ = 0.835, *T*_max_ = 1.000
10174 measured reflections
3247 independent reflections
2248 reflections with *I* > 2σ(*I*)
*R*_int_ = 0.071

#### Refinement

*R*[*F* ^2^ > 2σ(*F* ^2^)] = 0.046
*wR*(*F* ^2^) = 0.100
*S* = 1.00
3247 reflections
226 parameters
H-atom parameters constrained
Δρ_max_ = 0.89 e Å^−3^
Δρ_min_ = −0.66 e Å^−3^

### 

Data collection: *SMART* (Bruker, 2000 ▶); cell refinement: *SAINT* (Bruker, 2000 ▶); data reduction: *SAINT*; program(s) used to solve structure: *SHELXS97* (Sheldrick, 2008 ▶); program(s) used to refine structure: *SHELXL97* (Sheldrick, 2008 ▶); molecular graphics: *ORTEP-3* (Farrugia, 1997 ▶) and *PLATON* (Spek, 2009 ▶); software used to prepare material for publication: *SHELXL97*.

## Supplementary Material

Crystal structure: contains datablock(s) global. DOI: 10.1107/S1600536812000980/bt5782sup1.cif

Additional supplementary materials:  crystallographic information; 3D view; checkCIF report

## Figures and Tables

**Table:** 

Pd1—N3	2.059 (4)
Pd1—N4	2.039 (5)
Pd1—S1	2.3090 (17)
Pd1—S2	2.3045 (15)

**Table:** 

N4—Pd1—N3	86.73 (16)
S2—Pd1—S1	82.78 (6)

**Table 2:** Hydrogen-bond geometry (Å, °)

*D*—H⋯*A*	*D*—H	H⋯*A*	*D*⋯*A*	*D*—H⋯*A*
C12—H12⋯N2^i^	0.95	2.54	3.306 (7)	138
C14—H14⋯N5^ii^	0.95	2.49	3.277 (8)	140

Symmetry codes: (i) 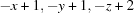; (ii) 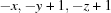.

**Fig. 1.**
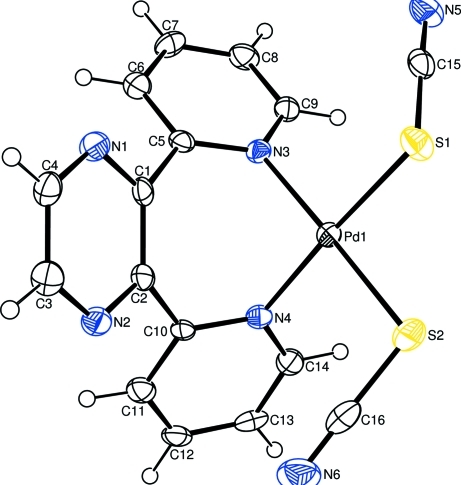
A view of the molecular structure of the title complex, with displacement ellipsoids drawn at the 40% probability level and the atom numbering.

**Fig. 2.**
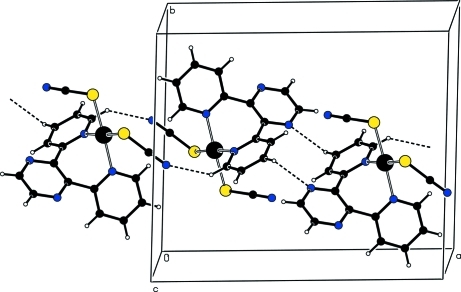
A partial view of the crystal packing of the title complex. Hydrogen-bond interactions are drawn with dashed lines.

## supplementary crystallographic information

### Comment

The title complex, [Pd(SCN)_2_(dpp)] (dpp = 2,3-di-2-pyridylpyrazine, C_14_H_10_N_4_), is isomorphous with the previously reported analogous halogen complexes [Pd*X*_2_(dpp)] (*X* = Cl, Br or I) (Ha, 2011*a*,*b*,*c*). The Pd^II^ ion is four-coordinated in a distorted square-planar environment by the two pyridine N atoms of the chelating dpp ligand and two S atoms from two thiocyanate anions (Fig. 1). The coordination mode of the dpp ligand is similar to that found in the mononuclear Pt(II) and Pd(II) complexes [PtCl_2_(dpq)] (dpq = 2,3-di-2-pyridylquinoxaline) (Granifo *et al.*, 2000) and [MCl_2_(dcdpp)] (*M* = Pt, Pd; dcdpp = 2,3-dicyano-5,6-di-2-pyridylpyrazine) (Cai *et al.*, 2009).

In the crystal, the pyridine rings are considerably inclined to the least-squares plane of the PdS_2_N_2_ unit [maximum deviation = 0.027 (1) Å], making dihedral angles of 70.3 (2)° and 69.2 (1)°. The nearly planar pyrazine ring [maximum deviation = 0.013 (4) Å] is almost perpendicular to the unit plane with a dihedral angle of 86.3 (1)°. The dihedral angle between the two pyridine rings is 76.8 (2)°. The thiocyanato ligands are located on opposite sides of the PdS_2_N_2_ unit plane and are almost linear with the bond angles of <S1—C15—N5 = 177.8 (6)° and <S2—C16—N6 = 178.9 (6)°. The Pd—N and Pd—S bond lengths are nearly equivalent, respectively (Table 1). The complex molecules are stacked in columns along the *b* axis and are connected by intermolecular C—H···N hydrogen bonds, forming chains along the *a* axis (Fig.2 and Table 2). In the columns, numerous inter- and intramolecular π-π interactions between the six-membered rings are present, the shortest ring centroid-centroid distance being 4.061 (3) Å.

### Experimental

To a suspension of Na_2_PdCl_4_ (0.1475 g, 0.501 mmol) and KSCN (0.5456 g, 5.614 mmol) in MeOH (30 ml) was added 2,3-di-2-pyridylpyrazine (0.1176 g, 0.502 mmol), and stirred for 24 h at room temperature. After removal of the formed brown precipitate by filtration, the solvent of the filtrate was evaporated. The residue was washed with H_2_O, and dried at 50 °C, to give a yellow powder (0.2119 g). Crystals suitable for X-ray analysis were obtained by slow evaporation from a CH_3_CN solution.

### Refinement

H atoms were positioned geometrically and allowed to ride on their respective parent atoms [C—H = 0.95 Å and *U*_iso_(H) = 1.2*U*_eq_(C)]. The highest peak (0.89 e Å^-3^) and the deepest hole (-0.66 e Å^-3^) in the difference Fourier map are located 1.05 Å and 0.99 Å from the atoms S1 and Pd1, respectively.

### Crystal data

**Table:**

[Pd(NCS)_2_(C_14_H_10_N_4_)]	*F*(000) = 904
*M**_r_* = 456.82	*D*_x_ = 1.828 Mg m^−^^3^
Monoclinic, *P*2_1_/*c*	Mo *K*α radiation, λ = 0.71073 Å
Hall symbol: -P 2ybc	Cell parameters from 2951 reflections
*a* = 15.8236 (11) Å	θ = 2.6–25.6°
*b* = 13.5901 (9) Å	µ = 1.38 mm^−^^1^
*c* = 7.9189 (6) Å	*T* = 200 K
β = 102.960 (1)°	Plate, yellow
*V* = 1659.5 (2) Å^3^	0.27 × 0.25 × 0.13 mm
*Z* = 4	

### Data collection

**Table:**

Bruker SMART 1000 CCD diffractometer	3247 independent reflections
Radiation source: fine-focus sealed tube	2248 reflections with *I* > 2σ(*I*)
graphite	*R*_int_ = 0.071
φ and ω scans	θ_max_ = 26.0°, θ_min_ = 2.0°
Absorption correction: multi-scan (*SADABS*; Bruker, 2000)	*h* = −19→19
*T*_min_ = 0.835, *T*_max_ = 1.000	*k* = −16→14
10174 measured reflections	*l* = −9→9

### Refinement

**Table:**

Refinement on *F*^2^	Primary atom site location: structure-invariant direct methods
Least-squares matrix: full	Secondary atom site location: difference Fourier map
*R*[*F*^2^ > 2σ(*F*^2^)] = 0.046	Hydrogen site location: inferred from neighbouring sites
*wR*(*F*^2^) = 0.100	H-atom parameters constrained
*S* = 1.00	*w* = 1/[σ^2^(*F*_o_^2^) + (0.0351*P*)^2^] where *P* = (*F*_o_^2^ + 2*F*_c_^2^)/3
3247 reflections	(Δ/σ)_max_ < 0.001
226 parameters	Δρ_max_ = 0.89 e Å^−^^3^
0 restraints	Δρ_min_ = −0.66 e Å^−^^3^

### Special details

**Table:**

Geometry. All e.s.d.'s (except the e.s.d. in the dihedral angle between two l.s. planes) are estimated using the full covariance matrix. The cell e.s.d.'s are taken into account individually in the estimation of e.s.d.'s in distances, angles and torsion angles; correlations between e.s.d.'s in cell parameters are only used when they are defined by crystal symmetry. An approximate (isotropic) treatment of cell e.s.d.'s is used for estimating e.s.d.'s involving l.s. planes.
Refinement. Refinement of *F*^2^ against ALL reflections. The weighted *R*-factor *wR* and goodness of fit *S* are based on *F*^2^, conventional *R*-factors *R* are based on *F*, with *F* set to zero for negative *F*^2^. The threshold expression of *F*^2^ > σ(*F*^2^) is used only for calculating *R*-factors(gt) *etc*. and is not relevant to the choice of reflections for refinement. *R*-factors based on *F*^2^ are statistically about twice as large as those based on *F*, and *R*- factors based on ALL data will be even larger.

### Fractional atomic coordinates and isotropic or equivalent isotropic displacement parameters (Å^2^)

**Table:**

	*x*	*y*	*z*	*U*_iso_*/*U*_eq_	
Pd1	0.19166 (3)	0.47138 (3)	0.53401 (6)	0.02445 (15)	
S1	0.11316 (10)	0.43246 (12)	0.2597 (2)	0.0379 (4)	
S2	0.23273 (12)	0.30866 (11)	0.5347 (2)	0.0455 (5)	
N1	0.3412 (3)	0.7055 (3)	0.3986 (6)	0.0299 (12)	
N2	0.4466 (3)	0.5833 (3)	0.6359 (6)	0.0298 (11)	
N3	0.1593 (3)	0.6182 (3)	0.5309 (5)	0.0229 (10)	
N4	0.2666 (3)	0.4960 (3)	0.7755 (6)	0.0236 (11)	
N5	−0.0358 (3)	0.5536 (4)	0.2015 (7)	0.0434 (14)	
N6	0.3992 (4)	0.3047 (4)	0.7615 (8)	0.0519 (16)	
C1	0.3097 (3)	0.6576 (4)	0.5210 (7)	0.0237 (13)	
C2	0.3637 (3)	0.5968 (4)	0.6413 (7)	0.0249 (13)	
C3	0.4761 (4)	0.6298 (4)	0.5124 (8)	0.0330 (15)	
H3	0.5350	0.6215	0.5063	0.040*	
C4	0.4231 (4)	0.6892 (4)	0.3940 (8)	0.0317 (14)	
H4	0.4459	0.7197	0.3058	0.038*	
C5	0.2195 (3)	0.6873 (4)	0.5241 (7)	0.0230 (13)	
C6	0.1985 (4)	0.7861 (4)	0.5149 (8)	0.0323 (14)	
H6	0.2410	0.8338	0.5051	0.039*	
C7	0.1161 (4)	0.8153 (4)	0.5200 (8)	0.0328 (15)	
H7	0.1013	0.8832	0.5138	0.039*	
C8	0.0551 (4)	0.7456 (4)	0.5339 (8)	0.0331 (14)	
H8	−0.0020	0.7645	0.5399	0.040*	
C9	0.0785 (4)	0.6479 (4)	0.5391 (7)	0.0256 (13)	
H9	0.0365	0.5995	0.5487	0.031*	
C10	0.3414 (3)	0.5479 (3)	0.7954 (7)	0.0218 (12)	
C11	0.3978 (4)	0.5543 (4)	0.9550 (7)	0.0298 (14)	
H11	0.4494	0.5921	0.9681	0.036*	
C12	0.3797 (4)	0.5061 (4)	1.0957 (8)	0.0308 (14)	
H12	0.4190	0.5086	1.2056	0.037*	
C13	0.3029 (4)	0.4541 (4)	1.0736 (8)	0.0323 (14)	
H13	0.2884	0.4206	1.1684	0.039*	
C14	0.2480 (4)	0.4517 (4)	0.9131 (8)	0.0292 (14)	
H14	0.1947	0.4171	0.8992	0.035*	
C15	0.0257 (4)	0.5056 (4)	0.2271 (8)	0.0326 (14)	
C16	0.3312 (5)	0.3072 (4)	0.6693 (9)	0.0371 (16)	

### Atomic displacement parameters (Å^2^)

**Table:**

	*U*^11^	*U*^22^	*U*^33^	*U*^12^	*U*^13^	*U*^23^
Pd1	0.0243 (3)	0.0191 (2)	0.0282 (3)	0.00066 (18)	0.00218 (18)	−0.00024 (19)
S1	0.0347 (10)	0.0434 (10)	0.0325 (10)	−0.0005 (7)	0.0006 (8)	−0.0074 (7)
S2	0.0538 (12)	0.0205 (8)	0.0555 (12)	0.0066 (7)	−0.0018 (9)	−0.0058 (7)
N1	0.029 (3)	0.028 (3)	0.034 (3)	0.002 (2)	0.009 (2)	0.004 (2)
N2	0.026 (3)	0.028 (3)	0.036 (3)	0.003 (2)	0.008 (2)	0.002 (2)
N3	0.027 (3)	0.022 (2)	0.020 (3)	−0.0008 (19)	0.005 (2)	0.0022 (19)
N4	0.022 (3)	0.022 (2)	0.027 (3)	0.0048 (18)	0.006 (2)	0.0001 (19)
N5	0.028 (3)	0.052 (4)	0.046 (4)	0.001 (3)	−0.002 (3)	0.004 (3)
N6	0.039 (4)	0.052 (4)	0.065 (5)	0.016 (3)	0.013 (3)	0.009 (3)
C1	0.021 (3)	0.019 (3)	0.029 (3)	−0.004 (2)	0.002 (3)	−0.007 (2)
C2	0.025 (3)	0.020 (3)	0.027 (3)	−0.002 (2)	0.000 (3)	−0.004 (2)
C3	0.029 (4)	0.032 (3)	0.042 (4)	0.003 (3)	0.014 (3)	−0.004 (3)
C4	0.033 (4)	0.024 (3)	0.040 (4)	−0.003 (3)	0.011 (3)	−0.002 (3)
C5	0.024 (3)	0.025 (3)	0.017 (3)	0.001 (2)	−0.001 (3)	0.001 (2)
C6	0.033 (4)	0.028 (3)	0.032 (4)	0.000 (3)	0.000 (3)	0.000 (3)
C7	0.043 (4)	0.019 (3)	0.037 (4)	0.009 (3)	0.010 (3)	0.002 (3)
C8	0.026 (3)	0.034 (3)	0.037 (4)	0.009 (3)	0.003 (3)	−0.001 (3)
C9	0.029 (3)	0.028 (3)	0.019 (3)	−0.004 (2)	0.004 (3)	0.001 (2)
C10	0.016 (3)	0.018 (3)	0.029 (3)	0.005 (2)	0.001 (2)	0.001 (2)
C11	0.027 (3)	0.027 (3)	0.034 (4)	0.006 (2)	0.004 (3)	−0.002 (3)
C12	0.029 (4)	0.026 (3)	0.032 (4)	0.011 (3)	−0.004 (3)	−0.001 (3)
C13	0.043 (4)	0.025 (3)	0.029 (4)	0.014 (3)	0.008 (3)	0.008 (3)
C14	0.023 (3)	0.029 (3)	0.037 (4)	0.003 (2)	0.010 (3)	0.002 (3)
C15	0.034 (4)	0.041 (4)	0.021 (3)	−0.014 (3)	0.002 (3)	0.003 (3)
C16	0.047 (4)	0.021 (3)	0.049 (5)	0.006 (3)	0.022 (4)	0.006 (3)

### Geometric parameters (Å, °)

**Table:**

Pd1—N3	2.059 (4)	C3—C4	1.372 (8)
Pd1—N4	2.039 (5)	C3—H3	0.9500
Pd1—S1	2.3090 (17)	C4—H4	0.9500
Pd1—S2	2.3045 (15)	C5—C6	1.382 (7)
S1—C15	1.677 (7)	C6—C7	1.371 (7)
S2—C16	1.679 (7)	C6—H6	0.9500
N1—C4	1.324 (7)	C7—C8	1.374 (8)
N1—C1	1.352 (7)	C7—H7	0.9500
N2—C3	1.333 (7)	C8—C9	1.377 (7)
N2—C2	1.336 (6)	C8—H8	0.9500
N3—C5	1.347 (6)	C9—H9	0.9500
N3—C9	1.356 (6)	C10—C11	1.378 (7)
N4—C14	1.334 (7)	C11—C12	1.377 (8)
N4—C10	1.356 (6)	C11—H11	0.9500
N5—C15	1.151 (7)	C12—C13	1.383 (8)
N6—C16	1.157 (8)	C12—H12	0.9500
C1—C2	1.398 (7)	C13—C14	1.369 (8)
C1—C5	1.488 (7)	C13—H13	0.9500
C2—C10	1.499 (7)	C14—H14	0.9500
			
N4—Pd1—N3	86.73 (16)	C6—C5—C1	118.9 (5)
N4—Pd1—S2	92.92 (12)	C7—C6—C5	120.0 (5)
N3—Pd1—S2	177.82 (13)	C7—C6—H6	120.0
N4—Pd1—S1	175.50 (12)	C5—C6—H6	120.0
N3—Pd1—S1	97.51 (13)	C6—C7—C8	119.4 (5)
S2—Pd1—S1	82.78 (6)	C6—C7—H7	120.3
C15—S1—Pd1	105.1 (2)	C8—C7—H7	120.3
C16—S2—Pd1	103.5 (2)	C7—C8—C9	118.6 (5)
C4—N1—C1	117.4 (5)	C7—C8—H8	120.7
C3—N2—C2	117.8 (5)	C9—C8—H8	120.7
C5—N3—C9	118.5 (4)	N3—C9—C8	122.4 (5)
C5—N3—Pd1	120.0 (4)	N3—C9—H9	118.8
C9—N3—Pd1	121.5 (3)	C8—C9—H9	118.8
C14—N4—C10	119.1 (5)	N4—C10—C11	120.6 (5)
C14—N4—Pd1	120.2 (4)	N4—C10—C2	119.7 (5)
C10—N4—Pd1	120.2 (4)	C11—C10—C2	119.8 (5)
N1—C1—C2	120.4 (5)	C12—C11—C10	120.2 (5)
N1—C1—C5	113.0 (5)	C12—C11—H11	119.9
C2—C1—C5	126.2 (5)	C10—C11—H11	119.9
N2—C2—C1	120.9 (5)	C11—C12—C13	118.5 (6)
N2—C2—C10	112.2 (5)	C11—C12—H12	120.8
C1—C2—C10	126.7 (5)	C13—C12—H12	120.8
N2—C3—C4	121.3 (5)	C14—C13—C12	119.1 (5)
N2—C3—H3	119.4	C14—C13—H13	120.4
C4—C3—H3	119.4	C12—C13—H13	120.4
N1—C4—C3	122.1 (5)	N4—C14—C13	122.4 (5)
N1—C4—H4	118.9	N4—C14—H14	118.8
C3—C4—H4	118.9	C13—C14—H14	118.8
N3—C5—C6	121.0 (5)	N5—C15—S1	177.8 (6)
N3—C5—C1	120.1 (5)	N6—C16—S2	178.9 (6)
			
N3—Pd1—S1—C15	−32.7 (2)	N1—C1—C5—N3	133.2 (5)
S2—Pd1—S1—C15	149.5 (2)	C2—C1—C5—N3	−54.5 (8)
N4—Pd1—S2—C16	−26.8 (3)	N1—C1—C5—C6	−44.9 (7)
S1—Pd1—S2—C16	151.9 (2)	C2—C1—C5—C6	127.3 (6)
N4—Pd1—N3—C5	68.5 (4)	N3—C5—C6—C7	2.8 (8)
S1—Pd1—N3—C5	−110.0 (4)	C1—C5—C6—C7	−179.1 (5)
N4—Pd1—N3—C9	−110.1 (4)	C5—C6—C7—C8	−0.1 (9)
S1—Pd1—N3—C9	71.4 (4)	C6—C7—C8—C9	−1.2 (9)
N3—Pd1—N4—C14	115.9 (4)	C5—N3—C9—C8	2.6 (8)
S2—Pd1—N4—C14	−66.3 (4)	Pd1—N3—C9—C8	−178.8 (4)
N3—Pd1—N4—C10	−72.1 (4)	C7—C8—C9—N3	0.0 (8)
S2—Pd1—N4—C10	105.8 (4)	C14—N4—C10—C11	0.4 (7)
C4—N1—C1—C2	2.5 (8)	Pd1—N4—C10—C11	−171.7 (4)
C4—N1—C1—C5	175.3 (5)	C14—N4—C10—C2	179.1 (4)
C3—N2—C2—C1	0.0 (8)	Pd1—N4—C10—C2	7.0 (6)
C3—N2—C2—C10	−174.9 (5)	N2—C2—C10—N4	−135.8 (5)
N1—C1—C2—N2	−1.2 (8)	C1—C2—C10—N4	49.7 (7)
C5—C1—C2—N2	−173.0 (5)	N2—C2—C10—C11	42.9 (6)
N1—C1—C2—C10	172.9 (5)	C1—C2—C10—C11	−131.6 (6)
C5—C1—C2—C10	1.2 (9)	N4—C10—C11—C12	1.5 (7)
C2—N2—C3—C4	−0.3 (8)	C2—C10—C11—C12	−177.2 (5)
C1—N1—C4—C3	−2.8 (8)	C10—C11—C12—C13	−1.9 (8)
N2—C3—C4—N1	1.7 (9)	C11—C12—C13—C14	0.5 (8)
C9—N3—C5—C6	−4.0 (8)	C10—N4—C14—C13	−1.9 (7)
Pd1—N3—C5—C6	177.4 (4)	Pd1—N4—C14—C13	170.2 (4)
C9—N3—C5—C1	177.9 (5)	C12—C13—C14—N4	1.4 (8)
Pd1—N3—C5—C1	−0.7 (7)		

### Hydrogen-bond geometry (Å, °)

**Table:**

*D*—H···*A*	*D*—H	H···*A*	*D*···*A*	*D*—H···*A*
C12—H12···N2^i^	0.95	2.54	3.306 (7)	138
C14—H14···N5^ii^	0.95	2.49	3.277 (8)	140

Symmetry codes: (i) −*x*+1, −*y*+1, −*z*+2; (ii) −*x*, −*y*+1, −*z*+1.
